# Hæmoglobinæmia Toxica

**Published:** 1884-10

**Authors:** 


### ART. XXVII.—HÆMOGLOBINÆMIA TOXICA.

("Azoturia."—*Williams.* "Hæmoglobinuria toxica."—*Bollinger.*)

We have been repeatedly importuned by readers of the JOURNAL for a contribution upon this very mysterious equine malady; but although we have had quite a number of cases, and have seen many others, the conditions of private practice are such as to have prevented us from making the necessary laboratorial researches, upon which one is only warranted in making any personal conclusions. For this reason, we think it far more in the interests of our readers to offer for their consideration a translation of the very elaborate paper of Prof. Bollinger * of Munich, upon the subject to which he has given the above name.

The German name is not "Schwaze Harnwinde," as given by Williams, but "Schwarze Harnwinde." The mistakes in the manner of spelling are at once self-evident. The translation which we offer is the only detailed consideration of the subject of which we know by a truly scientific observer. It has been

* Zeitschrift für Thiermedicin, vol. iii., p. 155.

universally accepted in Germany, and but little added to it by others since its first publication in 1877; still the subject is by no means a closed one, especially in connection with that rare complication, lameness of the vasti and rectus femoris of one or the other posterior extremities, which some American practitioners look upon as upward dislocation of the patella—an opinion we do not personally agree with. Bollinger speaks of the frequency of this disease in Germany, of its malignant course, and of the obscurity in which its etiology is still shrouded.

Great divergency of opinion rules among observers with reference to the real nature of the pathological processes, which consist principally in their sudden eruption and violent course, in the singular changes which occur in the constituency of the voice, and the paralytic condition of the posterior parts of the body. A trustworthy Boston practitioner has seen the latter confined almost entirely to the anterior limbs.

The views of authors can be, in general, divided into four classes, as follows:

I. That the ens of this malady is to be sought essentially in diseased processes located in the kidneys, which represent themselves as Morbus Brightii, acute paranchymatous nephritis, cloudy swelling, or hæmorrhagic nephritis.

(*a*) An intermediate position to the second is assumed by those who consider that coeval changes occur in the blood with the venal complications, and that the blood changes are to be looked upon as the cause of the pathological disturbances in the kidneys.

II. Those who look upon the ens of the disease to consist of changes in the blood—dyscrasis, dissolution analogous to those which occur in typhus-purpura, and anthrax. Others give it the name of lumbar-typhus, spinal-typhus, etc. Primary changes in the blood have been assumed by Spinola, Vogel, Haubner, and others. According to Siedamgrotzky, neither proto nor deuteropathic changes of this nature occur which can be connected with the processes in the muscles, kidneys, or produce hæmoglobinuria.

III. According to another group of observers, the primary cause is to be sought in pathological disturbances in the medulla spinalis, with deuteropathic paralysis of the venal nerves

—Franck, Sauer, and Fried; the latter differs, however, somewhat from the others in that he looks upon the protopathic condition as hyperæmia of the med. spinalis and its meninges, with deuteropathic venal disturbances. Related to the above are the views of those authors who look upon the conditions as rheumatic inflammation of the organs of the lumbo-sacral regions, or as congestions of the medulla and its surroundings and the intestines; others consider it to be due to toxic paralysis of the m. spinalis.

IV. A fourth view is that the entire malady is of rheumatic origin—which is just the same as saying that they know nothing about it.

None of these views offer anything very satisfactory to our consideration. In brief, the disease may be described as occurring very suddenly without any prodronal symptoms. A singular thing in Germany is that it is almost universally seen in heavy, phlegmatic work horses; while in Boston, every case that I have had (translator) has occurred in unusually well-bred animals; in fact, I can say, that though I saw a large number of cases while in the hospital at Berlin, I never saw it in a fine-bred animal.

It generally occurs after the animals have been stabled for a few days; the first symptom is generally labored movement, with violent perspiration, tottering in the posterior extremities, the animals falling frequently as if in a stroke of apoplexy, they are frequently unable to rise again. Soon one or the other posterior extremity may be paralyzed in a peculiar manner. To this we have previously alluded. As a rule, we may observe a very sudden and important œdematous tumefaction of the muscles of the croup and loins, which extends to those of the posterior portion of the back. These muscles seem as hard as boards. The urine soon suffers very marked changes; it becomes dark red, coffee, or chocolate colored, and has a strong acid reaction, at first often alcoholic; its specific gravity is high, and it contains, when microscopically examined, either none at all, or a very few red blood cells, characteristic albumen cylinders, which are at first hyalin, but later on are granulous or epithelial in character, and have been derived from the urinary tubes. Chemically examined, the urine contains large quantities of albumen. Williams denies this, but

I can confirm Bollinger's assertions from very many analyses of urine; in fact, it is pathologically impossible for albumen not to be present when we consider to what changes the condition of the urine is due. Hæmoglobin can be distinctly diagnosed by subjecting the urine to spectral analysis; hence the name.

Recovery frequently follows rapidly when the conditions are favorable. In violent cases we may observe a moderate exacerbation of the temperature, which generally follows the rule of being higher towards evening, 40–41° C.; this degree is sometimes seen in the earliest stages of the malady. Both pulse and respiration are increased. The appetite sometimes continues good; in others it is materially interfered with. Thirst generally increased considerably. Consciousness almost always undisturbed. In favorable cases the specific gravity of the urine diminishes rapidly, the albumen and dark color soon disappear, and in the course of twenty-four hours the recovery seems almost complete. Sometimes paralysis of one of the posterior extremities may continue for weeks or months. I have a case in which it has lasted five months, being no better now than when it first appeared; the muscles alluded to are in a condition of extreme atrophy, and the patient distressingly lame in the leg. An autopsy seems impossible on account of attachment on the owner's part, much as I should like to add its results to this paper.

Autopsical observation reveals the blood to be thickened, blackish; the affected muscles soft, looking as if cooked; the liver slate-colored; the kidneys softened; the bladder either nearly empty, or containing a thick, reddish fluid. The arachnoideal fluid in the spinal canal is often increased, and the vessels injected.

Case I.—Autopsy: Kidneys apparently hypertrophied, of a dirty brownish red color, and putrid degeneration begun, although the examination was made immediately on the death of the patient. It could not be decided whether the granular detritus found in the urine, *intra-vitam*, was due to paranchymatous degeneration in the kidneys or to this putrid dissolution. The medulla spinalis, in the lumbar region, was of a clouded dirty reddish color, but of normal consistency. The duration of the malady was about seven hours, yet the urine had the color of dirty Bordeaux wine, and when microscopically examined, contained granulous and albuminous molecules, the latter being frequently united in bunches, numerous micrococci, but neither cylinders or red blood cells.

Chemical analysis of the urine showed that it contained 1.18 per cent. of hæmoglobin.

Case II.—Autopsy: Acute degeneration and softening of the kidneys; extensive œdema of the adjacent parts, the m. spinalis and psoas muscles; liver hypertrophied, with granular degeneration; hæmorrhagic centres in the spleen and under the endocardium and pleuræ; blood very dark, and did not coagulate.

In special: On the surface of the left kidney one could see numerous stellate injections surrounded with small hæmorrhages into the venal substance. Corresponding to these external appearances, the cut surface of the kidney presented a peculiar spotted and streaked appearance; these variations were of a red color, sometimes wedge-shaped. The remaining parenchyma was very pale, anæmic, and had a moist lustrous surface.

Examined microscopically, the epithelium of urinary tubes was in a condition of extreme granular degeneration, clouded swelling; the hæmorrhagic centres betrayed a diffuse imbibition of the venal tissue, with red blood cells collected here and there in the tubes; in short, parenchymatous nephritis, with acute degeneration and circumscribed hæmorrhages.

The urine was the same as in Case 1; the ilio-psoas muscles were extremely œdematous, and in a condition of granular and hyaline degeneration, the continuity of the fibres being frequently broken. Other muscles showed similar changes.

Case III.—Numerous tests of the urine were made *intra-vitam*, all of which demonstrated the presence of extreme quantities of albumen, which, according to Tappeiner's examination, proved to be undisturbed hæmoglobin. The urine examined on the fourth day of the disease contained about double the quantity of this albuminous material to that of the first day. Microscopically examined, aside from micrococci, the urine contained quantities of granular detritus composed of molecules of albumen, also cylinders of like character, composed either of granules or epithelium ; no blood cells.

The clinical and patho-anatomical analysis of all the symptoms of this peculiar malady may be stated about as follows. The clinical representation gives us :

1. Hæmoglobinuria.
2. Albuminuria.
3. Paralysis or a paralytic condition of the posterior parts.

1st. Of these, hæmoglobinuria is the most important link in the chain. This symptom has generally been spoken of as "bloody urine"—hæmaturia—a nomenclature that does not exactly correspond to the facts, and which has given rise to many misconceptions. In simple cases of hæmaturia, due to a variety of causes, the urine is never of this peculiar color, and when microscopically examined, contains well characterized red blood cells in varying number. On the contrary, in hæmoglobinuria, the urine is of a marked and peculiar color, and when subjected to microscopic examination, contains in general no red blood cells; occasionally they have been seen, however, and have their origin with the albumen cylinders in deuteropathic disease of the kidneys ; the hæmoglobin can be easily demonstrated by spectral analysis. This element is one of the chief constituents of the red blood cells, and is an albuminoid which is bound on the coloring stuff of the blood ; it gives access to the urine by passing from the blood through the kidneys. The circumstance that the reddish color of the urine in this disease is not due to the presence of red blood cells, but to the excreted hæmoglobin, was first practically demonstrated by Franck. The same author has also shown that the red blood cells do not dissolve in urine ; hence the disturbance or dissolution of these cells must take place in the blood itself in this disease. This fact is necessarily followed by the conclusion that changes in the blood must constitute a primary movement in the pathology of this disease.

In order to arrive at a more secure basis for this hypothesis, it is necessary for us to discuss the question, "Under what analogous conditions do we also observe hæmoglobinuria?" (It should be interpolated here that the name hæmoglobinuria cannot be applied to the disease as such, but is rather applicable to a single but special symptom of the disease, as is also

Williams' nomenclature. When we know the whole train of circumstances in this strange disease, it will have to have another nomenclature.—TRANS.)

This malady is but little known in human medicine, although it occurs. It has, on the contrary, been studied experimentally, especially by Ponfick. In his experiments upon transfusion of blood, Ponfick found in his animals that he always produced hæmoglobinuria when he had introduced, transfused, a certain quantity of foreign blood. Such blood acted like a poison. It always underwent dissolution as soon as the colored elements—red cells—lost their vital characteristics. In this way hæmoglobin was set free, and was held in solution by the plasma, and in this condition and through this medium was brought in contact with all the tissues and organs of the body. A portion of the hæmoglobin suffers metamorphosis in the circulation, but the greater part is excreted by the kidneys, and thus becomes mixed with the urine. If the quantity of hæmoglobin is not too great, it can be excreted by the kidneys without difficulty, especially when their action is supported by an artificial diuresis. If the quantity of hæmoglobin is too great, however (0.5 per cent. of weight of the body of lamb's blood transfused into a dog), anatomical changes in the kidneys are the immediate result. In consequence of the violent inflammation that quickly develops in the kidneys, a profuse exudation in the urinary tubes soon follows, which at once renders further excretion of the hæmoglobin impossible, thus conducing to a literal termination.

If the quantity of transudated blood—i. e., blood from another animal—be less, then only a small part of the *tuberli uriniferi* becomes obstructed, or it is after the transudation of the hæmoglobin that inflammatory processes of great extension occur. In variable cases, where the excretion takes place more slowly and the general phenomena are more severe, the cylinders seem to be, as it were, washed out by a more profuse diuresis, thus giving a free course for the exudation of the hæmoglobin.

According to these facts, it seems justifiable to assume that the toxic action of unnatural blood is due to the sequential nephritis, and the consequent uræmia.

Hæmoglobinuria in horses distinctly proves that other organs may also become complicated, such as the posetic conditions produced in the muscles.

We shall presently quote evidence from Vait that certain materials in the digestive tract can also influence the production of hæmoglobinuria.

2d. *Albuminuria.* As is known, in the urine of horses suffering from this malady we not only find hæmoglobin in solution, but as a rule, also, cylinders of albumen, epithelium, and granular detritus. These cylinders distinctly indicate that the paranchyma of the kidneys must be diseased, desquamative nephritis, or morbus Brightii acutus. These results, which have been confirmed by microscopical observations, have led authors to look upon morbus Brightii as the ens of this malady, and parenchymatous nephritis as the most essential process.

Emphasizing the latter conditions did not, however, lead to any explanation as to the genesis of the hæmoglobinuria, which seems to have escaped due attention on account of the strong evidence offered by the presence of albumen and venal detritus in the urine. It has long been known with reference to acute Bright's, that in many severe cases venal hæmorrhage and the presence of red blood cells in the urine may be met with ; but protopathic albuminuria with a deuteropathic hæmoglobinuria has been thus far an unrecognized occurrence.

The previously quoted experimental evidence most clearly proves that through certain media—transfusion of foreign blood—we can produce hæmoglobinuria at will, and through the latter, also, acute Bright's. The question is, Are the processes in this equine malady of an approximate nature ?

As we shall show later on, desquamative parenchymatous nephritis can be caused not only by the increased pressure thus put upon the venal functions when dissolved hæmoglobin is suspended in the blood, but that that unknown quantity which causes hæmoglobinuria may also exert an irritative action upon the renal parenchyma, as well as upon that of the liver and muscles. It is therefore evident that inflammation of the kidneys ante-dates the presence of hæmoglobin as a soluble quantity in the blood. Still another important process occurs hand in hand with the acute nephritis, viz : with apoplectic suddenness appear the paretic conditions in the posterior extremities. This symptom, which often appears to be the one introducing the malady to our notice, and which has been almost generally attributed to complications of the medulla spinalis, has been looked upon by many authors as the primary seat of the disease; in proof of which we have only to quote the names of "kreuzlähme"—sacral lameness; "ruckenmarktypus"—typhus of m. spinalis; paraplegia, nervous paralysis, etc.

If this view were the correct one, it must self-evidently be demonstrated that the hypathetic affection of the m. spinalis was the protopathic lesion. The motor paralysis of the posterior extremities, as well as the renal complications, interposed by means of paralysis of the renal nerves, were supposed to be dependent upon the primary complication of the m. spinalis. Franck was one of the first to generalize these hypotheses, which were more or less fully assumed by Vogel and Friedberger. He considers the almost apoplectic paresis of the posterior parts of the body, which so frequently comes in the earliest moments of the disease, as speaking decidedly for the correctness of this view, and reports that in two such cases the artificially evacuated urine was at first completely normal, but in about an hour it was coffee colored and albuminous. To this it may be objected that the urine which was at first drawn was in the bladder before the disease appeared.

Vogel (Stuttgart), who has treated over 100 such patients during the last ten years, has generally observed the dark urine to be the first indication of disease, and that it always preceded the posterior paresis; in other cases, however, the paretic phenomena seemed to have introduced the disease, to be immediately followed by the excretion of urine pathognomonic of this malady. The condition of the bladder, the time after the first notice of disease at which the urine was tested, should all play an important role in deciding such a question.

Microscopical examinations have not as yet given us any proof of disease of the m. spinalis of such a nature as to satisfactorily explain the cause of the motor disturbances in the posterior parts. The results of such examinations simply show a hyperæmic condition of the meninges of the medulla spinalis, and the presence of an increased quantity of serum in the arachnoidial space. According to our physiological and pathological experiences, hyperæmic conditions of the m. spinalis always led to the presentation of irritative phenomena, so that in hyperæmia of the lumbar portion, convulsion, or at the worst, tetanus of the corresponding part results. Some of the spasmodic conditions observed in this disease can only find this explanation in such an assumption.

Another complication seems to me to play quite an essential role in the generation of the paralytic or paretic conditions of this disease. I mean the extensive œdema and the sequential degeneration of the muscle fibres in the internal and external muscles of the lumbo-sacral region, as can be demonstrated *intra-vitam.*

Corresponding to the microscopically easily demonstrated œdema of the tissues in the vicinity of the kidneys—the ilio-psoas muscles—we can microscopically show the presence of important changes in the parenchyma of the muscle-fibres which of itself is sufficient to account for the paretic phenomena. If we add to this the fact that the nerve-sheaths and the stem of the m. cruralis are also in the highest degree œdematous, it would appear that such changes were in themselves sufficient anatomical cause for the intra-vitally observed motor disturbances. Numerous observers have also reported similar œdematous conditions of the external muscles of these regions as almost constant phenomena. If, as the majority of observers have assumed, the m. spinalis is the original and primary seat of disease, then it would be most difficult to account for those paretic conditions which have been so frequently reported as being limited to only one of the posterior extremities. If these paretic conditions were due to m. spinalis complications, we should also be able to find disturbances of a sensitive as well as motor character—anæsthesia; further, uncontrolled passage of fœces and urine would be a natural consequence. But we find none of these conditions as existing, and the evidence certainly shows that we must look for the cause of the proplegia and lumbar paralysis in the œdematous conditions of the external and internal muscles of the lumbo-sacral region. It is self-evident that the possibility of collateral œdema of the lumbal m. spinalis is by no means denied; and coeval with the paresis, we may sometimes find anæsthetic conditions in the afflicted parts.

Some general remarks with reference to *paralysis of the pos-*

*terior parts in animals*, may not be out of place here. Such conditions have often been reported by practitioners as of no inconsiderable importance. While the causes of these disturbances have been generally sought in the central nervous system, they are in reality mostly of a peripheral nature, and to be considered as an expression of weakness. If it were demanded of very sick or very weak men that they must stand on all-fours, as animals do, we should just as frequently meet with paretic conditions of the posterior parts in man as in animals. It is not at once evident why it is in animals that posterior extremities refuse service sooner than the anterior; it may be that their greater distance from the heart has something to do with it.

If, according to the evidence, we look upon the ens of hæmoglobinuria to consist primarily in disturbances of the blood, which first of all lead to the setting free of hæmoglobin and the sequential production of hæmoglobinuria; and secondly, leads to the production of nephritis with albuminuria; and lastly, that paresis of the posterior parts is due to œdema of the lumbar muscles, and possibly of the m. spinalis also—we are still of the conviction that we have not said very much that is new.

Earlier observers have long ago reported that the venesected blood in this malady did not give a clear natural, but a reddish serum on coagulation, which Spinola attributed to the presence of dissolved hæmatin. At autopsies the blood was found to be black and viscid; the muscles soft, and looking as if they had been boiled. Microscopically examined, they presented the condition of fatty degeneration in a very ultra degree. Siedamgrotzky found the highest degree of molecular degeneration with interruption of the continuity of the parenchyma, in a large number of specimens. The liver is generally of slate or clay color, and somewhat softened. Franck places especial emphasis upon the hepatic complications in this disease as being a constant phenomenon. In such cases the microscopic examination of the cells of the liver reveals the fatty degeneration of their plamea, and their swollen contours—clouded swelling.

All these changes in connection with hæmoglobinuria sufficiently indicate that some toxa, or toxic element or material, must be looked upon as the cause of all the processes of this malady, which exerts its injurious action primarily upon the blood in the same manner that transudation of foreign blood also exerts a poisonous influence under certain conditions, leading to the production of hæmoglobinuria and nephritis. Hence this disease must also be classed with the *intoxication diseases*, although we do not know the nature of the poisonous element or elements at present.

We come now to the consideration of the most important and at the same time almost unanswerable question: *What causes the hæmoglobinuria?* Authors have assumed that cold exerted an influence upon it; but the sudden eruption which characterizes this malady is sufficient to contradict that assumption, as well as the fact that no amount of exposure to cold has ever been known to produce the succession of acute and marked phenomena which we see in this disease.

As easy as it is to demonstrate the impossibility of cold having any etiological connection with hæmoglobinuria, it is no less difficult to arrive at any understanding of the true cause, even though we become most positively convinced of its poisonous nature. For this hypothesis speak all the phenomena of the malady, most especially its apoplectoid eruption and violent course, which are not known to occur in connection with any primary affection of either the kidneys or m. spinalis from other causes. All the evidence directly contradicts the presence of an inficiens in this disease, so that we need not consider that point.

After giving all the points of cause a careful consideration, we are forced to the conclusion that the etiological moment must be introduced into the organism, or have some connection with the food. In this manner a toxic something is introduced or produced which causes a partial dissolution of the hæmoglobin, it being set free and held in solution in the blood-serum, hæmoglobinuria, and parenchymatous nephritis. In consequence of the extremely acute nature of the produced nephritis, it is not to be wondered at that collateral œdema of the adjacent tissues should soon follow, and consequently the most disturbances—paresis—of motor functions of the posterior groups of muscles.

The frequently observed fact of the eruption of this malady after the horses have had a day or two's rest, and then being put to work, apparently in perfect health, cannot at present be explained, only conjectured.

Since making the above translation, we have received the last issue of the "Archiv für Thierheilkunde," Berlin, which contains an article by Frœhner of Stuttgart upon the same subject. It is rather a peculiar and none the less satisfactory occurrence, i. e., to us, that he should have adopted at the same time the name *Hæmoglobinæmia* for this singular disturbance. We have long thought this should be the proper name, and have been surprised that it did not occur to Bollinger instead of that of "Hæmoglobinuria toxica," for the latter name but indicates one of the results and conditions; it is not the condition of the urine in the bladder which has any causal nexus with the trouble, but rather the changes which occur in the blood, though we do not yet know what influence or cause sets the hæmoglobin free from the red corpuscles.

Could we but know this, we could then more correctly name the disease. But as we cannot at present get back to the first cause, and as the freed hæmoglobin seems to be the active toxic principle that causes the principal phenomenon with which we are acquainted, it is but a logical conclusion that this should be the name of the disease. Williams' name, "Azoturia," is of but little meaning, save that which custom has given it, and has the same objections that "Hæmoglobinuria" has; it simply means a superabundance of urates in the urine, which, as has been shown, is not the chief condition of the urine by any means. Frœhner considers the malady to be primarily of a rheumatic nature—that is, it is due to the influence of cold upon the animals after being confined in the hot air of the stable; but it will be more just to him to let him speak for himself:

"How can cold generate hæmoglobinæmia?" "Numerous influences are known to be able to produce hæmoglobinæmia, most of them being of a chemical nature. Of these are gallic acid—Kühne; water injected into the veins—Herman; the same of glycerine—Luchsinger; sulphuric acid—Naunyn; muriatic acid—Vogel; arsenated hydrogen—Voit; nitro-benzol—Bœhm; iodine—Neisser; pyro-gallic acid—Marchaud; chlorate of potash—Ellenberger and Hoffmeister; copper poisoning—Ponfick; blood transfusion of another species; following severe burns, etc. In all these cases we must assume that disturbance of the red blood cells must have been the cause of the freedom of the hæmoglobin (and its suspension as a free albuminate in the blood serum). Can we transfer these observations or conclusions directly to the equine malady under consideration? Not so!"

"It is indeed known that blood assumes a lac color after freezing, in consequence of the dissolution of the red blood cells; but the animals receive no such exposure to cold in these cases, even in their superficial capillaries."

"My ideas of the influence of cold in the production of hæmoglobinæmia are as follows: It is a well recognized physiological fact that metamorphosis of the elements of the muscles is accelerated through irritation of the sensitive temperature nerves of the skin. These changes first occur in the fat and circulating albumen, and if the irritation is increased, extend to the organ albumen or plasma of the muscle cells, leading to degeneration of the fibres and stacking of the circulating blood, with sequential œdema of the same tissues. In this manner we can explain the muscle-changes found in horses at autopsies."

"Among the products of the metamorphosis thus occurring in the muscles, and which are taken up by the blood, is the coloring stuff of the muscles themselves, aside from the urates, etc. This material is chemically identical with hæmoglobin. As a proof of this substance being set free, we refer to the well known pale, washed-out appearance of the affected muscles, which is of so extreme a degree as to warrant the conclusion that the coloring stuff is absent; and when we ask where, the only answer is, *taken up by the circulation.* In mild cases these changes are so slight that we are unable to find any hæmoglobin in the urine. No matter what the quantity is that is thus freed from the muscles, it gets into the circulation, and is to be looked upon as a foreign material, and must be eliminated by the excretory organs."

"In these cases do we indeed have to do with so powerful an irritant? It seems to be a well constituted fact that the disease occurs in horses after confinement in the stable, which are often extremely warm and illy ventilated, and that after having adapted themselves to such a temperature, the effect of exposure to the cold air of the external world must have an extreme effect."

So much for the views of others.

We do not accept Frœhner's "Catching Cold" theory:

1st. Because the disease often occurs here in Boston in the hot months of July and August.

2d. All the horses that are kept in do not have it, as will be seen in the following remarks.

Every one who has had much experience in this disease knows that it will, or does, occur frequently in one horse of a team that are of nearly the same age, that have exactly the same work to do daily, that have the same feed, the same water, the same care. They rest over Sundays or perhaps two days—for this thing never has been reported in horses without rest—they have their usual feed on working days; they go out on Monday, all feeling gay as larks; go a half mile; one breaks into a perspiration, totters and falls, and is hardly able, sometimes unable, to get to the stable. The urine at once being drawn, is characteristic. Diagnosis is made. We ask ourselves why this one horse and not the other. Here the unknown primary quantity is the *predisposing cause.* In my personal experience I have had my driving horses forced to twelve or fourteen quarts of grain a day, and often left them in their boxes for two weeks at a time; taken them out and driven them pretty hard, yet I never had anything of this kind occur.

The past spring a patron drove a small trotting mare two miles into Boston, and on account of rain coming on, did not drive her out for two days; she was sick on getting back home, and with that peculiar form, paresis of one hind leg, especially the vasti, etc. This mare had been accustomed to just such rests, even for weeks, and high feeding, then sudden drives. The question is, *Why this malady this time*, and never before?

It will be at once seen that we do not want any more teaching schools in the world half so much as we need some inaugurated for research. In fact, nearly every disease which we have been asked to write upon lately is etiologically unknown, and all the result which our labors have shown is that, like ourselves, veterinary authors know little or nothing save symptoms and treatment, and some suggestive practical observations.—TRANS.

